# Impact of an early educational protocol on the oral language of children born preterm exhibiting phonological fragility: a multicenter randomized clinical trial

**DOI:** 10.3389/fpsyg.2024.1393246

**Published:** 2024-12-05

**Authors:** Aude Charollais, Vincent Laudenbach, Marie-Hélène Stumpf, Benoît Delaporte, Valérie Datin-Dorriere, Thierry Debillon, Claire De Barace, Olivier Flechelles, Marie Farmer

**Affiliations:** ^1^LAMOPRESCO research group, University Rouen Hospital, Rouen, France; ^2^Service of Pediatric, MFME University Hospital, Fort de France Martinique, France; ^3^Research center on psychological functions and dysfunctions, EA7475, University of Rouen, Mont Saint-Aignan Cedex, Rouen, France; ^4^Service of Neonatology and Neonatal intensive Care Charles Nicolle University Hospital, Rouen, Cedex, France; ^5^Service of Neonatology and Neonatal intensive Care, Jacques Monod Hospital, Le Havre Cedex, France; ^6^Service of Neonatology and Neonatal intensive Care, Côte de Nacre Caen Normandie University Hospital, Caen Cedex 09, France; ^7^Université de Paris, CNRS UMR 8240, Sorbonne University, Paris, France; ^8^Neonatal Intensive Care Unit, Grenoble Alpes University Hospital, La Tronche, Grenoble, France; ^9^FMSS Sherbrooke University Qβc, Sherbrooke QC, Canada

**Keywords:** prematurity, oral language disorder, language and motor skills, school-age, child born preterm, phonology score, education speaking

## Abstract

We conducted a six-center, prospective, randomized, open-label trial to assess whether an early standardized educational protocol provided from 42 to 48 months of age improved the progression of oral language and phonological development in children born preterm. A total of 552 children with phonological fragility were included in this study. The children were randomized to receive the educational protocol (guided arm, *n* = 87) or not (non-guided arm, *n* = 78). In the guided arm, the oral language development used a short “say and do” type educational protocol designed to maintain visual attention and train the developmental phonology/lexicon/morphosyntax structural links. In contrast, a conservative approach was used in the non-guided arm. A total of 70 guided and 73 non-guided children completed the study. After 6 months, the educated children showed a non-significant increase in their phonology score (*p* = 0.37), while the variations in the scores of the expressive lexicon (secondary endpoints) were significantly improved (*p* = 0.0008). We conclude that the short, standardized stimulation of the sensorimotor aspects of language in children born very preterm increased the expressive lexicon. This protocol improved the language of the premature children, especially those with minimal motor skills, with more significant improvement in the phonological scores.

**Clinical trial registration:**

clinicaltrials.gov, identifier NCT01426659

## 1 Introduction

The Epipage 1 study (Etude épidémiologique sur les Petits âges GEstationnels, epidemiological study on small gestational ages-1) showed a decreasing rate of cerebral palsy in very preterm newborns when compared to historical series (Fily et al., 2006; Marret et al., 2007). However, the Epipage 2 study reported a high frequency of minor neurological difficulties (Ages and Stages Questionnaire [ASQ] score less than −2 standard deviations in 42.7 to 55.8% of infants at 2 years of age) (Pierrat et al., 2017). School failure, often related to learning difficulties such as learning disorders(including dyslexia, the inability to read) and phonological fragility (a delay in the automation of reading that does not fall within the scope of pathology) (see Methods), is overrepresented in children born preterm or very preterm compared to children born at term (Larroque et al., 2011; Martinez-Nadal and Bosch, 2021). This also includes difficulties regarding the mastery of writing.

Evidence of the benefits of early intervention in these children to prevent learning disabilities (reading, writing, and arithmetic) remains limited. Some studies have shown a benefit in acting early in terms of education in this population, but they only evaluated the overall effects (intellectual efficiency, i.e., intelligence quotient) (Kelly et al., 2020) or were conducted on relatively small sample sizes, yielding contradictory results (Zuccarini et al., 2020). A recent meta-analysis showed that early intervention programs for preterm infants positively influence cognitive and motor outcomes during infancy, with cognitive benefits persisting until preschool age. However, there is much heterogeneity between the studies due to the variety of early developmental intervention programs tested and the gestational ages of the preterm infants included, making comparisons of the intervention programs challenging (Spittle et al., 2015). Therefore, further research is needed, as recommended by Spittle et al. (2012), to determine which early developmental interventions are most effective at improving cognitive and motor outcomes.

*The first interactions between the different components of language certainly do not have the same impact on the acquisition of the lexicon.*

Phonological development predicts reading performance in premature children (Wocadlo and Rieger, 2007). Furthermore, there is a time window during early development, at the end of which the phonological component of oral language must be in place; otherwise, it is unstable and dysfunctional (Kuhl, 2010). The development of phonology is based on neurodevelopmental prerequisites, including motor skills (Bates, 1992). Articulatory “gesture” motor skills serve as a unit of phonological contrast and characterization of the resulting articulatory movements (Namasivayam et al., 2020). The motor theory of speech perception was not conceived from a developmental viewpoint (Galantucci et al., 2006). However, if it corresponds to a neurophysiological organization of the brain, then does it exist precociously in infants, and can it help us understand the phonological vulnerability of children born prematurely with many other minor motor and praxis disorders? Phonology 7,5 mois (Kuhl 2008) in the mother tongue through oral-facial motor expressions within concomitant verbal and nonverbal human interactions, including fine motor skills. Moreover, according to Baddeley (Macken et al., 2014), the quality of phonology plays an essential role in working memory, and we May question its role when early global memory stimulation seems ineffective. One hypothesis could be that phonology can only be trained early with a lexicon to improve memory. Studies on co-development of the lexicon and phonology seem to be in agreement. Indeed, Anderson et al. recently found no significant effect of a working memory educational protocol applied for a few weeks at 2, 12, and 24 months in children born significantly premature, aged 5–7 years, prompting an evaluation of the benefit of earlier actions (Anderson et al., 2018). We hypothesized that in a population of children born prematurely, before 33 weeks of gestation, presenting phonological fragility (but not dysphasia), we would observe minimal motor disorders, including sensorimotor prerequisites of language that could hinder the development of phonology (i.e., the organization of sounds during speech). This hypothesis was in agreement with the role of production in representing the speech of infants and young children studied by Vihman (2022). The lexicon and phonology co-develop in interaction (Davis and DER Feest, 2018), making it possible to retain lexical items and “open up” phonological skills. The construction of phonological memory could depend on the quality of production (Vihman, 2022). If a minimal motor deficit interferes with early phonological production accuracy, the lexicon could be constrained. Verbal memory would be less developed early and would in turn hinder the development of the phonological knowledge required for reading, which is the organization of sounds during reading, and during a specific time window, cognitive stimulation adapted to particular sensorimotor stages can promote the development of the phonological component of language.

The phonological assessment must be conducted in the mother tongue. Furthermore, no study has analyzed the impact of a re-education protocol on the phonological component of the French language [a semi-transparent language spoken by approximately 300 million individuals worldwide (Beck et al., 2018)]. Therefore, we sought to demonstrate the effect of an early, short, and standardized educational protocol during a specific time window. For instance, before the age of 4, it would improve the phonological component of a score developed for the French language, the “BILO Petits” score (Bilan Informatisé de Langage Oral) used to clarify the difficulties of this population (Charollais et al., 2010). We also characterized other fragile or defective subtle sensorimotor elements in this study population, with subtle sensorimotor disorders affecting oral production directly and/or indirectly (see below Methods - Basis - Study population), bearing in mind that this is a population for whom routine and conventional speech therapy is not recommended by the official 2018 recommendations of the French Haute Autorité de Santé (HAS, n.d.). Thus, our main objective was to measure the effects of a short and standardized rehabilitation protocol on language components, including phonology and motor components.

## 2 Materials and methods

This study was conducted according to the French laws (Toulouse et al., 2018) and the ethical principles of the Declaration of Helsinki, and it was approved by the local ethics review committee (CPP Nord-Ouest I) before any research activity (decision made on 01/13/2011). The study was registered at the ClinicalTrials.gov website under NCT01426659.

### 2.1 Participants

Children met the inclusion criteria if they were 3.5 years old at the time of inclusion and were born before 33 weeks of gestation. Their parents signed a free and informed consent form. The children and their parents were affiliated with the national health insurance system. After being evaluated for oral language, they were defined as phonologically fragile or having atypical phonology (see “Baseline” below).

Children were excluded from the study if they met any of the following exclusion criteria: severe karyotype abnormality, any malformation of the central nervous system diagnosed prenatally or during the neonatal period (by transcutaneous ultrasound and/or magnetic resonance imaging), cerebral palsy, a persistent neurological disorder (e.g., acquisition of walking over 24 months), a major neurosensory disorder (e.g., blindness or profound deafness), ear-nose-throat or ophthalmological conditions, and guardianship issues.

### 2.2 Study design and overview

The LAMOPRESCO study (LAngage et MOtricité du PRÉmaturé d’âge SCOlaire, i.e., language and motor skills in the school-age child formerly born preterm) was a multicenter, prospective, randomized, open-label, and interventional study. The participants were recruited from the following six French clinical centers: Rouen University Hospital, Caen University Hospital, Grenoble University Hospital, Le Havre General Hospital, Tours Regional University Hospital, and St Brieuc General Hospital.

The eligible children were randomly assigned in a 1:1 ratio to either receive the educational protocol or a conservative approach. Block randomization with stratification was carried out at the clinical center by drawing a sealed opaque envelope at each randomization. At the baseline visit (V1), the children, 3 years and 6 months old (+/− 2 months), born very prematurely, and who were followed-up annually in one of the participating centers, underwent this first screening visit with one associate investigator to ensure that they met the recruitment criteria. Assessments of different skills were performed, and the results were collected by a pediatrician and a speech therapist trained in monitoring premature infants (see also below, “Endpoints”).

The “BILO Petits” test (Bilan Informatisé de Langage Oral, Computerized Oral Language Assessment for Toddlers) was administered. This 5-item score test in the French language evaluates (i) lexicon scores in reception and production, (ii) oral understanding, (iii) word repetition and statements based on a sequence of words, (iv) morphosyntax levels, and (v) comprehension level, which varies from 0 to 12 between 42 and 48 months of age (Charollais et al., 2010). The NEEL (Nouvelles Epreuves d’Examen du Langage, i.e., New Tests for Language Examination), which evaluates working memory and verbal planning (Plaza et al., 2002), a neuromotor examination, the Kaufman Assessment Battery for Children (K-ABC) (Kaufman et al., 1987), and the NEPSY (Bilan NeuroPSYchologique de l’enfant, i.e., Child Neuropsychological Evaluation) in its French version (Ahmad and Warriner, 2001) were also conducted to rule out any cognitive impairment. An evaluation of the child’s sensorimotor (i.e., pre-reading skills: visual attention, auditory attention, and static and dynamic oral facial praxis) and tactile constraints (evaluation of graphic gestures, tactile discrimination, and transfer of sensory modalities) was conducted using the COntraintes Sensitivo MOtrices Sensorimotor constraints (COSMO) test battery (Charollais et al., 2010).

Based on the scores obtained during the “BILO Petits” test, the children were divided into three groups: (i) a “normal” group, with a score of at least four out of five items above the 25th percentile and the fifth criterion being greater or equal to the 10th percentile; (ii) a fragile group, with either two items under the 25th percentile but over the third percentile, 1 item under the 10th percentile but above the third percentile, or an isolated value of the ProdE (expressive lexicon) of 0; and (iii) a “pathological” group, with at least one item under the third percentile (except for ProdE alone since, whatever its value, it never defines pathology by itself).

The children from the “normal” and “pathological” groups were excluded. The children with pathological oral language disorders were referred for classical speech therapy. Then, the eligible children in the fragile group were randomly assigned to one of the two following arms: with rehabilitation or without rehabilitation through a balanced distribution of interventions by sealed opaque envelopes. Block randomization with stratification was used by the clinical center.

### 2.3 Intervention arm

In the rehabilitation group, 22 consecutive speech therapy education sessions, designed for the French language [“Dire et Faire” (“say and do”) protocol (Charollais et al., 2010), and a speech therapy program, comparable to the Hanen “More Than Words” method (Carter et al., 2011)], were scheduled, with a 30-min weekly session for a period of approximately 6 months period (20 sessions).

This rehabilitation program was carried out by a speech therapist trained in conducting BILO at the investigation centers or at the speech therapist’s usual work site.

The “say and do” work diagram included three parts:

- Phonology with imitation: It involves asking the child to name pictures by slowing down the flow of words until segmentation begins. It involves naming the images with the adult, then repeating each word in slow motion while asking the child to fix their gaze on the adult’s mouth and say the word at the same time as the adult, for example, in French: “CHAt CHApeau CHApiteau,” and in English IPA: “/ʃa ʃapo ʃapito/.”
- Lexicon with a morphosyntactic aim (production of statements): The vocabulary is understood as a semantic component with functional, event-based, and categorical matches. For example, the child is made to look at pictures representing objects [names, events (verbs), scenes, scripts, and pictorial narratives] and is asked, “What is it used for?” For example, the child is shown a helicopter and asked to say what it does.
- Morphosyntax: The goal is to use temporal and causal relationships with boards, in which the child is asked what happened before or after the scene represented. For example,the child is shown a boy on the ground crying and asked what could have happened before and what the boy will do afterwards.

After each session, the parents were offered “homemade pictures” to practice for 5 min daily in the family setting or in a childcare center or kindergarten when the family could not meet this request.

### 2.4 Control arm

The control group benefited from a standard parental guidance approach, a combination of interventions that could be performed at home, such as repeating words or reading stories, along with encouraging the children to slow down their speaking rate. Speech and language therapy assessments at the beginning (3.5 years old) and at the end of the study (4 years old) were carried out by an independent assessor, a speech and language therapist, different from the one who conducted the rehabilitation sessions, to ensure a single-anonymized system and result validity.

### 2.5 Outcome measures

The primary outcome was the extent of the variation in the BILO Petits phonology score between the first and the last study visits (correct word repetition) in each arm (with or without rehabilitation) (Charollais et al., 2010). Therefore, the primary endpoint measure was defined as the difference between the BILO score at the baseline and the BILO score at the end of the study. Secondary endpoints were the extent of the variation in the BILO score for other components (reception and production lexicon, production of statements, and oral comprehension) (Charollais et al., 2010).

Similarly, the extent of variation in the following scores was measured by five different batteries at 42 months. The NEEL scores explored the semantic academic content of the language (Plaza et al., 2002) but did not assess the motor skills, unlike BILO. The NEPSY scores (Ahmad and Warriner, 2001) in its French version and the K-ABC scale (Kaufman et al., 1987) measured the cognitive and intellectual development. The motor measures were assessed by the BHK according to the psychomotor coordination scale, based on a BHK subtest adapted for the children aged 3.5 years (Gargot et al., 2020). The fine motor skills were assessed through the quality of programming and the execution of the graphic gesture and laterality scores according to the Edinburgh scale (Oldfield, 1971). The sensorimotor constraints scores were assessed using the COSMO battery.

Many somatic parameters interfering with “general” development were collected, including respiratory scores. (Kherkheulidze et al., 2018). The respiratory status was evaluated using the respiratory score to classify the children into three classes: (i) no respiratory problems, (ii) occasional mild bronchitis, and (iii) asthma, prolonged inhalation therapy, and prolonged physiotherapy.

### 2.6 Statistical analysis

The data were described at baseline, and 22 weekly visit sessions for all patients were included and separated by experimental groups, using the usual descriptive parameters: mean and standard deviation for the quantitative variables and frequencies and percentages for the qualitative variables. The differences in the means between the two groups and their 95% confidence intervals (CIs) were estimated.

A comparative intention-to-treat (ITT) analysis was performed according to the randomized rehabilitation arm.

The primary analysis was conducted among the intention-to-treat (ITT) population, which included patients who were randomized, according to the rehabilitation group drawn randomly. The ITT population was defined as all patients randomized in the study, regardless of whether the primary outcome was observed. The mean difference in the extent of variation in the BILO phonology score (word repetition) between 42 and 48 months of age was compared between the treated (educated) and non-treated (non-educated) arms using Student’s *t*-test. This analysis was based on the assumption of maximal bias, according to which the lowest observed improvement in the primary outcome was attributed to the children with missing primary outcomes in the treated arm. In contrast, the most significant improvement was attributed to the children with missing primary outcomes in the untreated arm. This crude comparison was complemented by an adjusted comparison based on the linear model and a generalized estimation equation approach to account for the correlations between twins. Adjustment covariates were centered (Rouen University Hospital vs. five other centers) because the randomization was stratified by the center, respiratory score, and the following covariates related to the primary outcome: laterality, microcephalus, and the score for graphic gestures.

We conducted two sensitivity analyses. The first *post hoc* sensitivity analysis was a full-case analysis that only included the children whose primary outcomes were not missing. In the second sensitivity analysis, multiple imputations were performed for the primary outcome, assuming that this primary outcome was missing at random, using a regression analysis based on participants with complete data, which relied on the following variables: center, treatment arm, laterality, respiratory score, microcephaly, initial phonology score, and graphic gesture score.

Each variable’s mean and standard error were estimated by generating 50 imputed sets using the MIANALYZE procedure, assuming a multivariate normal distribution of the data. The same approach as that described for the primary efficacy endpoint univariate analysis was used for comparisons relating to the four other BILO Petits score dimensions (lexicon in reception and production, oral comprehension, and statement production) and the NEPSY score by, once again, considering, for each criterion, the difference between the score obtained on the test post-rehabilitation and the score obtained on the initial test.

All statistical tests were two-sided, and a two-tailed significance level of 0.05 was used to establish statistical significance. The Bonferroni correction was applied to multiple comparisons of the secondary outcomes (the phonology score with lexicon scores in reception and production, oral understanding and production of statements, the NEEL and a neuromotor examination, and the K-ABC).

Statistical analyses were conducted using the SAS statistical software (version 9.4; SAS Institute; Cary, NC, United States).

### 2.7 Power analysis sample size calculation

Based on the preliminary data observed in two consecutive personal patient series, we assumed that we would observe a mean difference of at least 1.9 units in the BILO score variation (which varies from 0 to 16), corresponding to an effect size of 0.404 between the ages of 42 and 48 months. The sample size was calculated based on the Student’s *t*-test with a two-sided significance level of 0.05 to ensure 90% power for an effect size of 0.404. A total of 260 children were required (130 per arm).

## 3 Results

### 3.1 Study sample

**Figure 1:**
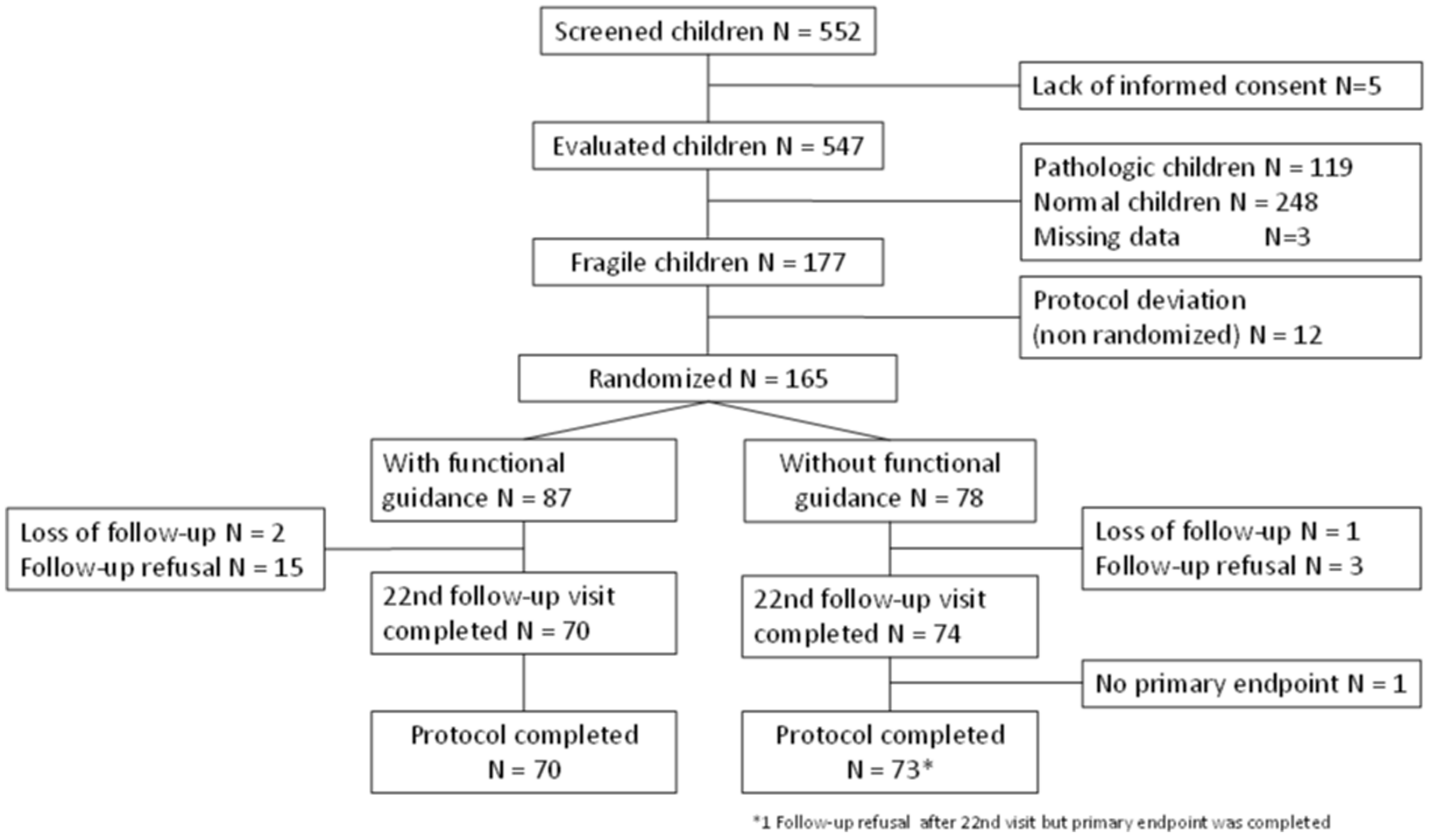
Flow chart: LAMOPRESCO.

Overall, 552 children who were referred to our tertiary care center between 31 August 31 2011 and 29 August 2014, including five whose parents refused participation, were screened (see Figure 1). The follow-up ended on 27 February 2015. Five children who did not provide informed consent were excluded. Among the 547 screened children, 177 were classified as “fragile,” but only 165 participants of a mean age of 3.6 ± 0.1 years were randomized (fragile group), which is 63.5% of the target sample size. Of the 165 randomized participants (87 in the rehabilitation group and 78 in the group without rehabilitation), the primary endpoint was available in 86.7% of the cases (143/165): 70/87 (80.5%) in the rehabilitation arm and 73/78 (93.6%) in the control arm.

The main characteristics of the 22 children with no follow-up visit at 6 months were described overall and by the rehabilitation group to ensure the absence of any major selection bias. The two groups had no significant differences in the baseline demographic and clinical characteristics (Table 1). Furthermore, there was no difference between the groups in the BILO phonology score at baseline, with mean scores of 7.8 ± 3.3 and 7.7 ± 3.5, respectively (*p* = 0.82, Table 2).

**Table 1:** Characteristics at baseline.

	With guidance *n* = 87	With no guidance *n* = 78	Total *n* = 165	*p*-value
Sex				0.700
Female	45 (51.7)	38 (48.7)	83 (50.3)	
Male	42 (48.3)	40 (51.3)	82 (49.7)	
Age at first visit (V1)				0.517
Years mean ± SD	3.6 ± 0.1	3.6 ± 0.1	3.6 ± 0.1	
Weight				0.065
*n*	86	76	162	
Mean ± SD (kg)	14.3 ± 2.3	13.7 ± 1.8	14 ± 2.1	
Height				0.400
*n*	86	76	162	
Mean ± SD	96.3 ± 4.6	95.7 ± 4.2	96 ± 4.4	
Gestational age				0.059
< 27 weeks of gestation	3 (3.4)	6 (7.7)	9 (5.5)	
27 to 29 weeks of gestation	24 (27.6)	32 (41)	56 (33.9)	
30 to 32 weeks of gestation	60 (69)	40 (51.3)	100 (60.6)	
Center				0.972
Caen	19 (21.8)	16 (20.5)	35 (21.2)	
Grenoble	9 (10.3)	7 (9)	16 (9.7)	
Le Havre	9 (10.3)	9 (11.5)	18 (10.9)	
Rouen	39 (44.8)	36 (46.2)	75 (45.5)	
St Brieuc	7 (8)	8 (10.3)	15 (9.1)	
Tours	4 (4.6)	2 (2.6)	6 (3.6)	
IUGR				0.859
No	71 (81.6)	62 (80.5)	133 (81.1)	
Yes	16 (18.4)	15 (19.5)	31 (18.9)	
Hyaline membrane disease				0.123
No	45 (51.7)	31 (39.7)	76 (46.1)	
Yes	42 (48.3)	47 (60.3)	89 (53.9)	
Chronic lung disease (CLD) with oxygen dependency				0.089
No	73 (83.9)	57 (73.1)	130 (78.8)	
Yes	14 (16.1)	21 (26.9)	35 (21.2)	
SCRESP				0.703
No respiratory problem	51 (58.6)	48 (61.5)	99 (60)	
Occasional or mild bronchitis or asthma; prolonged inhaled treatment, prolonged physiotherapy	36 (41.4)	30 (38.5)	66 (40)	
Microcephaly				0.349
No	74 (88.1)	63 (82.9)	137 (85.6)	
Yes	10 (11.9)	13 (17.1)	23 (14.4)	
Laterality				0.353
Right	70 (80.5)	67 (85.9)	137 (83)	
Left	17 (19.5)	11 (14.1)	28 (17)	
Graphic gesture evaluation				0.750
Score mean ± SD	13.8 ± 6.6	13.5 ± 6.8	13.6 ± 6.6	

CLD, Chronic Lung Disease; EEG, Electroencephalogram; IUGR, Intra Uterine Growth Retardation; MRI, Magnetic Resonance Imaging. *p*-values are the results of the Student’s *t*-tests or Chi^2^ tests; the results in bold are significant at the threshold of 0.05.

**Table 2:** Results of the amplitude of the variation of the BILO phonology score (word repetition) between 42 and 48 months of age.

Words repetition score /16	With educational protocol (*n* = 87)	Without educational protocol (*n* = 78)	Comparison^a^ mean difference / OR [95%CI]; *p*	Multivariate Comparison^b^ mean difference/OR [IC95%]; *p*
Baseline (V1) (*n* = 165)	*n* = 87	*n* = 78		
Mean (SD)	7.8 (3.3)	7.7 (3.5)	0.12 [−0.93; 1.18]; *p* = 0.820	
Post-treatment (after six-month follow-up) (*n* = 143)	*n* = 70	*n* = 73		
Mean (SD)	11.4 (2.8)	10.9 (3.5)	0.48 [−0.58; 1.54]; *p* = 0.374	
Worst-case scenario analysis (*n* = 165)				
The amplitude of the variation of the BILO phonology score	*n* = 87	*n* = 78		
Mean (SD)	2.8 (3.6)	3.8 (3.8)	−0.99 [−2.13; 0.15]; *p* = **0.088**	−0.87 [−1.9; 0.1]; *p* = **0.094**
Complete-case analysis (*n* = 143)				
The amplitude of the variation in the BILO phonology score				
Mean (SD)	4.0 (3.1)	3.3 (3.4)	0.67 [−0.39; 1.73]; *p* = 0.215	0.78 [−0.09; 1.65]; *p* = 0.077
Multiple imputation analysis (*n* = 165)c				
The amplitude of the variation in the BILO phonology score				
Mean (SD)	4.0 (3.1)	3.3 (3.4)		0.78 [−0.1; 1.6]; *p* = 0.0729

a *P*-values from a generalized linear model (GLM), a generalization of ordinary linear regression (Student’s *t*-test *p*-value).

b *p-values are the results of a log-binomial model using a generalized estimation equation approach to account for correlations between twins after adjusting for prognostic variables: center, microcephalia, laterality, respiratory score, graphic gesture, and initial BILO Phonology Score; the results in bold are significant at the threshold of 0.05.*

Univariate analysis was performed using a log-binomial model with a generalized estimation equation approach to account for correlations between twins, as described in the statistical analysis section, to compare the mean difference in the word repetition deltas between the first (V1) and 22nd visits (V22).

The extent of variation in the BILO phonology score between V1 and V22 (Words Repetition, WRep) differed according to laterality (3.9 ± 3.2 for the right-handed children vs. 2.3 ± 3 for the left-handed children, *p* = 0.011) and according to microcephaly (3.4 ± 3.2 for the normal children vs. 5.2 ± 2.9 for those with microcephaly, *p* = 0.011). It also depended on the initial value of the phonology score (slope f(x) = Delta Word Rep Score − 0.52 +/− 0.07, *p* < 0.0001) and the graphic gesture or fine motor skills score (slope f(x) = −0.09 +/− 0.04, *p* = 0.0280). The latter was the only sensorimotor constraint of the COSMO battery. No significant correlation was found for oral-facial praxis (*p* = 0.089), dynamic praxis (*p* = 0.106), static praxis (*p* = 0.177), tactile discrimination (somesthesia, *p* = 0.699), visual attention (*p* = 0.327), or intermodal transfer quality (*p* = 0.502).

After an adjusted comparison based on the same model, the results were more or less the same, with a mean difference in the word repetition deltas of −1.4 ± 0.58, *p* = 0.016 for laterality, 1.5 ± 0.55, *p* = 0.008 for microcephaly, slopes of −0.48 +/− 0.06, *p* < 0.0001 for the initial value of the phonology score, and − 0.08 +/− 0.04, *p* = 0.038 for the graphic gestures/fine motor skills score. No significant correlation was found for the center (*p* = 0.079) or respiratory score (*p* = 0.341).

### 3.2 Primary endpoint

**Figure 2:**
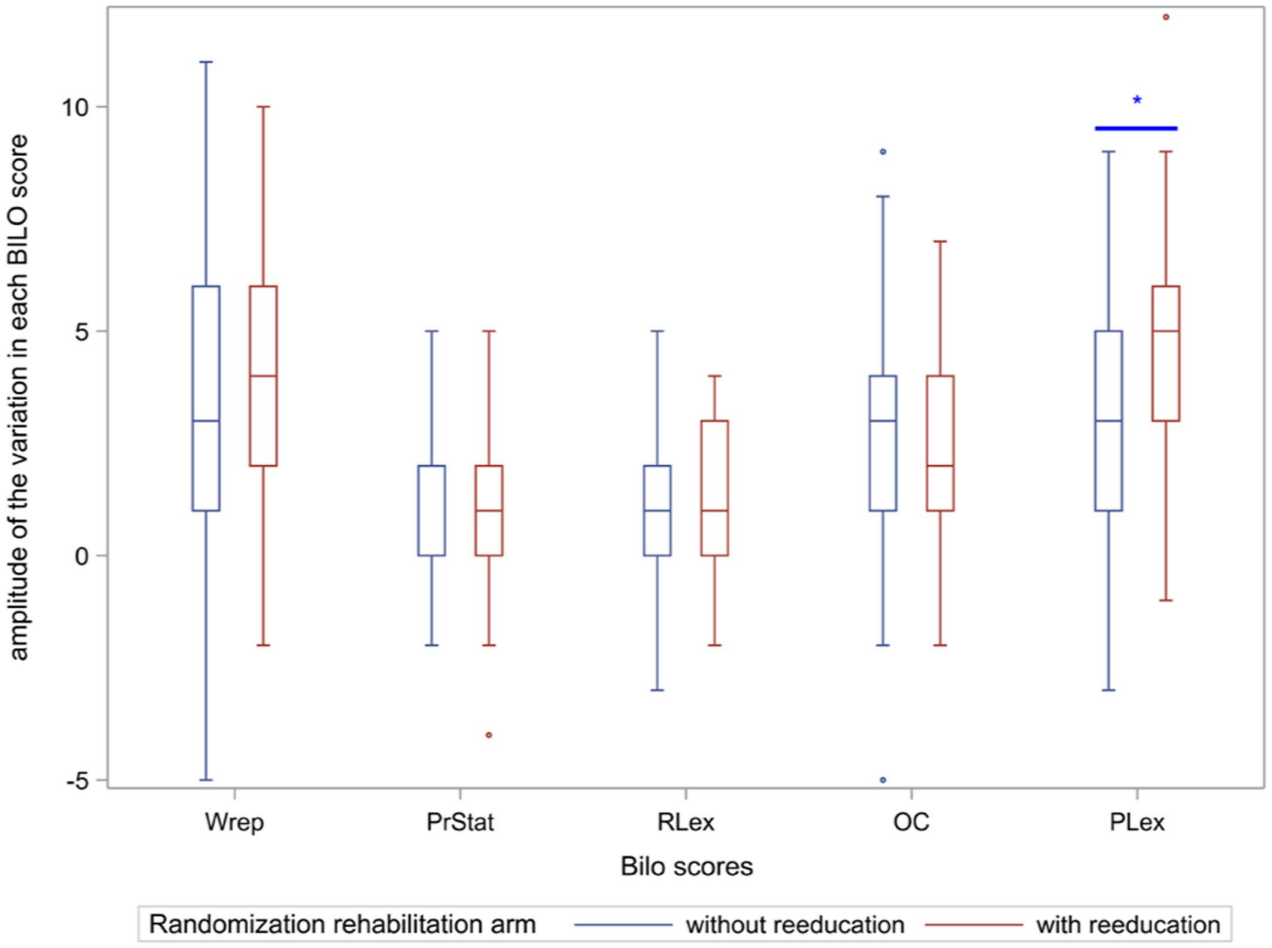
Variation between the first (V1) and the 22nd visit (V22) of the BILO score of the five components with and without rehabilitation. Wrep = Words repetition score, PrStat = Production of statements score, RLex = Reception lexicon score, OC = Oral comprehension score, PLex = Production lexicon score. *: *p* < 0.05; **: *p* < 0.01.

The primary endpoint, the BILO phonology score (word repetition), increased in both groups between the first (V1) and 22nd (V22) visits, and after 6 months, the educated children showed a non-statistically significant increase in the phonology scores (*p* = 0.37). However, there was a more significant improvement and more critical progress for the group with guidance. The difference in progress was significant (11.4 ± 2.8 compared to 7.8 ± 3.3 (mean ± SD, guidance) vs. 10.9 ± 3.5 compared to 7.7 ± 3.5 (mean ± SD, no guidance), with a mean difference of word repetition deltas of −0.99 ± 0.58, *p* = 0.09 for the worst-case scenario analysis or 0.67 ± 0.54, *p* = 0.22 for the complete-case analysis) (Table 2 and Figure 2). These results remained unchanged after adjusting for prognostic variables in both analyses: −0.87 ± 0.52 (*p* = 0.09) for the worst-case scenario analysis and 0.78 ± 0.44 (*p* = 0.08) for the complete-case analysis. Moreover, with the multiple imputation approach, the results were similar to those of the complete-case analysis, with an estimated mean difference of 0.78 ± 0.43 (*p* = 0.07) in the word repetition deltas.

### 3.3 Secondary endpoints

The score variation in the oral production lexicon of the BILO battery (secondary endpoints) was significantly improved: 15.9 ± 2.2 versus 11.1 ± 2.4 (guidance, V1 to V22) compared to 14.6 ± 2.5 versus 11.6 ± 2.7 (no guidance, V1 to V22), with a mean difference of oral production lexicon deltas of (1.66 [0.97; 2.35]), *p* < 0.001 (4.8 ± 2.4 vs. 2.9 ± 2.8 with and without guidance, respectively) (Table 2 and Figure 2). The differences in the score variations in the reception lexicon, oral comprehension, and statement production were not significant between the two groups: *p* = 0.556, *p* = 1, and *p* = 1, respectively, after the Bonferroni adjustment (Table 2 and Figure 2).

All six components of the NEEL (phonology, syntactic comprehension, topology, expression or vocabulary, verbal memory, and understanding or lexicon) typically increased in both groups between V1 and V22, without any significant difference between the two groups, except for the expression or vocabulary skill with a score variation mean of 6.5 ± 3.7 (guidance) compared to 4.4 ± 4.3 (no guidance), *p* = 0.032 (Table 3). The K-ABC II (psychological evaluation) and NEPSY scores also increased in both groups, but their differences were not statistically significant (Table 4).

**Table 3:** Results of the NEEL (Nouvelles Epreuves pour l’Examen du Langage) (New Tests for the Language Examination) (Anderson et al., 2018) at visit 1 (V1) and 22 (V22).

Results of the NEEL	Total (*n* = 165)	Delta (V22 - V1 scores) With guidance (*n* = 70)	Delta (V22 - V1 scores) With no guidance (*n* = 73)	*p* ^a^
Test 1: Phonology words	136	8.9 [8.0]	6.8 [9.0]	1
Test 2: Syntactic understanding list A	140	0.7 [1.2]	0.5 [1.2]	1
Test 2: Syntactic understanding list B	140	1.1 [1]	0.9 [1.1]	1
Test 3: Topology 1	140	3.4 [2.8]	2.4 [2.8]	0.328
Test 3: Topology 2 + 3	137	6.9 [5.4]	7.4 [5.9]	1
Test 4: Expression - Vocabulary	136	6.5 [3.7]	4.4 [4.3]	0.032
Test 5: Verbal Memory	124	7.3 [4.9]	6.1 [5.8]	1
Test 6: Comprehension - Lexicon	136	6.3 [4.1]	4.6 [4.1]	0.16

Values are expressed as mean [SD]. Bonferroni correction was applied. Results refer to subjects with complete data.

**Table 4:** Results of the K-ABC II (Vihman, 2022) and the NEPSY (Bilan NeuroPSYchologique de l’enfant, Child Neuropsychological Evaluation, 2nd edition) (Davis and DER Feest, 2018) scores at visit 1 (V1) and 22 (V22).

Results of the K-ABC II (Psychological evaluation) and the NEPSY scores	(*n* = 165)	Delta (V22 - V1 scores) with guidance (*n* = 70)	Delta (V22 - V1 scores) with no guidance (*n* = 73)	*p*^a^
Associative memory	137	15.4 [16.5]	16.5 [17.2]	1
Conceptual reasoning	137	3.7 [4.5]	3.6 [4.1]	1
Face recognition	135	3.5 [3.9]	2.2 [4.6]	0.624
Triangles	139	3.1 [2.5]	2.7 [2.6]	1
Word sequences	126	1.4 [2.7]	0.7 [2.1]	0.792
Denomination	137	3.4 [2.4]	3.2 [3.0]	1
Riddles	131	2.5 [2.1]	2.2 [2.8]	1
NEPSY	135	8.4 [5.8]	8.9 [5.7]	1

Values are expressed as mean ± SD. * The *p-*value was adjusted on eight tests according to the Bonferroni correction method.

a *P-values are the results of the Student’s t-tests; the results in bold are significant at the threshold of 0.05.*

## 4 Discussion

In this open-label, randomized controlled trial, we showed that an early, simple, and protocolized intervention on the language of 3.5-year-old children born very preterm and exhibiting phonological fragility (i.e., non-normally speaking because of various peculiarities, e.g., perseverations, facilitations, omissions, inversions, substitutions, without dysphasia) was effective for a population of patients for whom speech therapy is not routinely recommended, according to the current guidelines of the French High Health Authority released in 2018 (HAS, n.d.). This educational speech protocol, “say and do,” improves some components of oral language more than simple parental guidance. Although the difference between the two arms did not reach statistical significance for the primary endpoint, we observed a trend in the progression of the word repetition score (primary endpoint reflecting phonology) under the influence of the rehabilitation protocol. This trend was even more notable as their scores were low and they presented with motor characteristics. It should be noted that a larger-than-expected number of prematurely born children had to be excluded from the study as they presented with a real oral language disorder.

## 5 Study limitations

The number of children lost to follow-up, for whom the primary outcome could not be assessed, was high in the intervention group. Of the 22 children lost to follow-up out of 165 included, 17 belonged to the “intervention arm,” representing a 20% rate of missing primary outcomes, which were replaced by unfavorable values (as described in the methodology section on the intention-to-treat analysis). This was the worst-case scenario. Given that such differential attrition could distort the results of this study, we compared the sociodemographic and clinical characteristics of the children lost to follow-up with those who completed the study.

The children lost to follow-up were not significantly different from those analyzed for most baseline characteristics. However, they differed in two characteristics: lower phonological fragility scores in oral language development and fewer respiratory problems. One possible explanation is that the children least affected by these problems tended to drop out of the trial, particularly when they belonged to the intervention group, given the frequency of sessions and the perceived potential benefits. Conversely, the children with respiratory problems were more likely to attend consultations if their parents expected them to improve.

Moreover, the multiple imputation analysis supported these results because the results of the two analyses were very similar. However, we showed for the first time in this population that the amplitude of word repetition scores from the baseline to the end of the study was correlated with (i) graphic gesture quality and (ii) laterality. Notably, for the graphic gesture quality, the worse the initial value obtained by the children, the more they improved their phonological scores, according to the currently debated theory of motor speech perception (Axel, 2022). Choi et al. recently demonstrated that certain orofacial motor stages of language development preceding the onset of babbling are critical for developing phonological discrimination (Choi et al., 2021) within the motor prerequisites.

Regarding laterality, the left-handed children benefited significantly less from the educational protocol than the right-handed children. This finding was consistent with (i) the recently demonstrated overrepresentation of left-handedness among children born preterm (Marlow et al., 2019) and (ii) the already known higher frequency of left-handedness among children with dysphasia (Goez and Zelnik, 2008). Moreover, regarding the secondary endpoints, we showed that 22 weekly speech therapy sessions of 30 min each significantly improved word production (LexP) (*p* < 0.001). Although the differences in the score variations in the reception lexicon, oral comprehension, and statement production were not statistically significant, we observed a trend toward better progression for each skill under the influence of the re-educational protocol.

Similarly, for the components of the NEEL, K-ABC II, and NEPSY scores, a trend of better progression under the educational protocol influence was also observed, with a difference in the level of improvement between the two groups for the expression-vocabulary skill of the NEEL test (*p* = 0.032), in favor of the treated group.

This study has some limitations. First, it is important to note that the expected sample size could not be reached within the maximum period initially defined. This was primarily due to a higher proportion of pathological individuals in our cohort than expected [119/547, i.e., 21.8% (see Figure 1, flowchart)] when compared to the literature at the time of conducting the study (Harris et al., 2013). Therefore, this trial, which included 143 children (70 with active rehabilitation vs. 73 with no intervention), led to a power of 67% for the primary outcome analysis (instead of the expected 90%) for detecting a mean difference of at least 1.9 units in the BILO score variation.

Second, many patients were lost to follow-up (approximately 20 and 6% in the treated and control groups, respectively). Notably, the children in the intervention group started with slightly better potential, and their rehabilitation sessions helped them progress, which possibly led their parents to stop coming to their children’s consultations.

Third, the recruitment of the participating centers was heterogeneous. However, we compared the evolution of the children taken into care between the coordinating center and the other centers and all centers combined. No significant difference was observed, with mean phonology score variations between the initial and final value of 3.4 ± 3.3 in Rouen vs. 3.8 ± 3.2 in the other five centers, with a mean difference of 0.80 ([−0.09; 1.69], *p* = 0.079), which could have reduced a potential center-related recruitment bias (see also Table 5, Supplementary data).

**Table 5:** Quantitative variables associated with the difference in means of the word repetition score between V0 and V22 (Sco RepM).

*x*	*n*	Slope	The standard deviation of the slope	*p*^a^
		f(x) = Delta Sco RepM		
Sco_RepM at V1 (*n*=)	143			
Mean [SD]	7,5 [3.3]	−0,51	0,07	<0,0001
Evaluation of motor constraints				
Buccofacial praxis	141			
Mean [SD]	11.0 [4.1]	−0.13	0.08	**0.0889**
Dynamic praxis	143			
Mean [SD]	6.5 [2.5]	−0.2	0.12	0.1059
Static praxis	141			
Mean [SD]	4.5 [2.3]	−0.16	0.12	0.1772
Quality of the graphic gesture	143			
Mean [SD]	13.4 [6.6]	−0.09	0.04	**0.0280**
Tactile discrimination (somesthesia)	139			
Mean [SD]	6 [2.5]	−0.04	0.09	0.6994
Tactile discrimination (right hand)	140			
Mean [SD]	3.0 [1.4]	−0.16	0.17	0.3422
Tactile discrimination (left hand)	139			
Mean [SD]	3 [1.4]	0.07	0.17	0.6796
Visual attention	141			
Mean [SD]	4.7 [2.7]	−0.09	0.09	0.3266
Quality of the intermodal transfer	139			
Mean [SD]	5.2 [2.1]	−0.08	0.12	0.5019

Slope f (x) = Delta Sco RepM = Value of the slope of the improvement of the word repetition score between the first (V1) and the 22nd visit (V22).

a *P-values are the results of a log-binomial model using a generalized estimation equation approach to account for correlations between twins; the results in bold are significant at the threshold of 0.05.*

Fourth, we did not find any significant differences between the groups when using another oral language assessment system, the New Language Tests (NEEL), which is known to be less sensitive than the BILO test because it only evaluates the semantic content of the language and not the motor skills (Plaza et al., 2002).

Fifth, we wanted to test whether minimal motor disorders could predict the extent of improvement in the phonology word repetition component of the BILO score (WRep), a hypothesis raised by previous groups of researchers that is still controversial (Frome et al., 2020; Wolke et al., 2008). Notably, we found a correlation between the improvement in the word repetition score (phonology) and the quality of the graphic gesture but not with other praxes. This could be explained by the fact that the other praxes (buccofacial, static, and dynamic) develop earlier (Mary Lauren Neel et al., 2019) and are, therefore, mainly automated by 42 months, preventing them from progressing naturally at this stage of development without active speech therapy.

Despite these limitations, we have shown for the first time in a randomized trial that a proactive, short, inexpensive, and easy-to-implement phonological education protocol can help non-pathological premature infants who are identified as phonologically fragile and are not covered by systematic care recommendations in their language development, particularly when they present an associated vulnerability of graphic gestures.

To the best of our knowledge, this is the only demonstration focusing on a population of non-pathological and phonologically fragile children within a specific time window of language development. Furthermore, we highlighted that our intervention was aimed at children aged 3 years. This is a relatively late intervention age compared to recently published studies concerning other populations of children with learning disorders. However, our results suggest possibility of readily effective therapy intervention from compulsory schooling at age 3 in France. Furthermore, no results from a study, which evaluated an early phonological education protocol applied to very premature newborns, were reported, despite the characterization of numerous constraints (Charollais et al., 2013; Stipdonk et al., 2020) and better understandings (Bastianello et al., 2022). It would be interesting to apply a similar approach to children belonging to populations speaking opaque (e.g., English and Scandinavian) or semi-transparent languages other than French. Thus, the link between motor constraints and phonology could be universal, regardless of the phonotactic motor difficulty of the native language. Rehabilitation is recommended for better-defined categories of children.

## 6 Conclusion

We showed that brief and protocolized stimulation of the sensorimotor aspects of language between 3.5 and 4 years of age in children born prematurely with phonological fragility but without dysphasia led to a specific improvement in language skills. Interestingly, although 3.5 years is already a late age for intervention in the practice necessary for language development, specific sensorimotor stimulation of oral language can improve phonology, especially since there are vulnerabilities in fine and graphic motor skills. Therefore, we conclude that taking care of the motor components of phonology, even at school age, is likely to be beneficial for children. This confirms the current knowledge and research on the interactions between early sensorimotor development and the quality of language development. Early intervention at 18 months May provide more significant benefits.

## Data Availability

The datasets presented in this study can be found in online repositories. The names of the repository/repositories and accession number(s) can be found below: Trial registration The protocol has been declared to the Agence Nationale de Sécurité du Médicament (French National Drug Agency) under the number 2010-A00880-39 and registered on clinicaltrials.gov under the number NCT01426659.
